# A Novel Cryptic Three-Way Translocation t(2;9;18)(p23.2;p21.3;q21.33) with Deletion of Tumor Suppressor Genes in 9p21.3 and 13q14 in a T-Cell Acute Lymphoblastic Leukemia

**DOI:** 10.1155/2014/357123

**Published:** 2014-10-08

**Authors:** Moneeb A. K. Othman, Martina Rincic, Joana B. Melo, Isabel M. Carreira, Eyad Alhourani, Friederike Hunstig, Anita Glaser, Thomas Liehr

**Affiliations:** ^1^Jena University Hospital, Friedrich Schiller University, Institute of Human Genetics, Kollegiengasse 10, 07743 Jena, Germany; ^2^Croatian Institute of Brain Research, Salata 12, 10000 Zagreb, Croatia; ^3^Laboratory of Cytogenetics and Genomics, Faculty of Medicine, University of Coimbra, Azinhaga Santa Comba, Polo Ciências da Saúde, 3000-548 Coimbra, Portugal; ^4^Centro de Investigação em Meio Ambiente, Genética e Oncobiologia (CIMAGO), Rua Larga, 3004-504 Coimbra, Portugal; ^5^Jena University Hospital, Friedrich Schiller University, Department of Internal Medicine II (Oncology and Hematology), 07749 Jena, Germany

## Abstract

Acute leukemia often presents with pure chromosomal resolution; thus, aberrations may not be detected by banding cytogenetics. Here, a case of 26-year-old male diagnosed with T-cell acute lymphoblastic leukemia (T-ALL) and a normal karyotype after standard GTG-banding was studied retrospectively in detail by molecular cytogenetic and molecular approaches. Besides fluorescence in situ hybridization (FISH), multiplex ligation-dependent probe amplification (MLPA) and high resolution array-comparative genomic hybridization (aCGH) were applied. Thus, cryptic chromosomal aberrations not observed before were detected: three chromosomes were involved in a cytogenetically balanced occurring translocation t(2;9;18)(p23.2;p21.3;q21.33). Besides a translocation t(10;14)(q24;q11) was identified, an aberration known to be common in T-ALL. Due to the three-way translocation deletion of tumor suppressor genes *CDKN2A/INK4A/p16, CDKN2B/INK4B/p15*, and *MTAP/ARF/p14* in 9p21.3 took place. Additionally *RB1* in 13q14 was deleted. This patient, considered to have a normal karyotype after low resolution banding cytogenetics, was treated according to general protocol of anticancer therapy (ALL-BFM 95).

## 1. Introduction

T-cell acute lymphoblastic leukemia (T-ALL) is a quite rare and heterogeneous disease, more common in males than in females. It accounts for 15% of childhood and 25% of adult ALL cases [1]. Underlying genetic causes of T-ALL are poorly understood and this is highlighted by the fact that T-ALL is associated with a normal karyotype in 30–50% of the cases [2, 3]. In abnormal karyotypes recurrent chromosomal aberrations are reported [4]. Regularly, promoter and enhancer elements of genes involved in T-cell development are juxtaposed with translocations in close proximity of oncogenes [5, 6]. The most common structural chromosomal abnormalities in T-ALL are TCR (T-cell receptor) loci rearrangements. Breakpoints in 14q11 (TCRA/D) and 7q34 (TCR*β*) are observed frequently. Besides, deletions in the long arm of chromosome 6 may be found; the common deleted region involves mainly subband 6q16; however, candidate gene(s) have not been formally identified yet [7, 8]. Also tumor suppressor genes have been seen to be involved in T-ALL [9].

Cryptic structural chromosomal abnormalities are still a challenge in cytogenetic standard diagnostics of acute leukemia. However, many cryptic aberrations have been identified by molecular cytogenetics already. Examples in T-ALL are cryptic deletions in 9p21 involving the genes* CDKN2A/INK4A/p16*,* CDKN2B/INK4B/p15*, and* MTAP/ARF/p14* leading to loss of G1 checkpoint control of the cell cycle or the* RB1* locus in 13q14, which also plays a role as tumor suppressor gene in cell cycle regulation [9].

Here, a case of a young adult T-ALL patient with a novel cryptic three-way translocation, a reciprocal translocation, and submicroscopic deletions is reported.

## 2. Material and Methods

### 2.1. Clinical Description

A 26-year-old male presented in 1998 initially with a total white blood cell count of 20.2 × 10^9^/L, hemoglobin of 9.2 mmol/L, and platelets of 126 × 10^9^/L. Bone marrow examination was consistent with T-ALL having 91% blast cells. According to flow cytometry the immunophenotype of bone marrow lymphocytes was as follows: the cells were positive for CD2 (96%), CD8 (96%), CD4 (92%), CD7 (92%), CD1A (89%), CD10 (87%), CyCD3 (86%), and TdT (85%) and negative for *α*F1, *β*F1, CD3, CD13, CD19, CD20, CD24, CD33, CD34, HLA-DR, MPO-7, slg, TZR-*α*/*β*, and TZR*γ*/*δ*. The patient was treated according to ALL-BFM 95 protocol and died eight months after initial diagnosis from serious infections and severe complications while being in complete hematological remission.

### 2.2. Test Done at Diagnosis

GTG-banding was done according to standard procedures. A total of 7 metaphases were available for cytogenetic evolution derived from unstimulated bone marrow of the patient and were analyzed on a banding level of 180–250 bands per haploid karyotype [11] and determined as 46,XY [7, 12]. RT-PCR performed for* TEL/AML1* and* BCR/ABL* fusion genes was reported to be negative and fluorescence in situ hybridization (FISH) analysis carried out according to manufacturer's instructions for the same loci was negative (probes used: LSI BCR/ABL and LSI TEL/AML1, Abbott Molecular/Vysis, Mannheim, Germany).

### 2.3. Test Done in Retrospective

#### 2.3.1. Molecular Cytogenetics

FISH was done according to standard procedures and manufacturer's instructions for the following commercially available probes: LSI 13 in 13q14.2 (*RB1*, Abbott Molecular/Vysis, Mannheim, Germany), LSI IGH/BCL2 (*IGH* in 14q32;* BCL2* in 18q21, Abbott Molecular/Vysis, Mannheim, Germany), SPEC ALK/2q11 (*ALK* in 2p23, Zytovision GmbH, Bremerhaven, Germany), SPEC p16/CEN9 (p16 in 9p21.3, Zytovision GmbH, Bremerhaven, Germany), SPEC BIRC3/MALT1 (*BIRC3* in 11q22.2,* MALT1* in 18q21.32, Zytovision, Bremerhaven, Germany), and POSEIDON MLL/MLLT3 (*MLL* in 11q23.3,* MLLT3* in 9p21.3; Kreatech Diagnostics, Amsterdam, Netherland).

Whole chromosome painting (WCP) probe for chromosomes 2, 9, 10, 14, and 18 and bacterial artificial chromosome probes (BACs) for chromosomes 2 and 9 (Table 1) were homemade [13]. The homemade multitude multicolor-banding (mMCB) and chromosome specific high resolution array-proven multicolor-banding (aMCB) probe sets were also applied as previously reported [10, 14, 15].

A total of 10–15 metaphase spreads were analyzed, using a fluorescence microscope (AxioImager.Z1 mot, Zeiss) equipped with appropriate filter sets to discriminate between a maximum of five fluorochromes and the counterstain DAPI (Diaminophenylindol). Image capturing and processing were carried out using an ISIS imaging system (MetaSystems, Altlußheim, Germany).

#### 2.3.2. DNA Isolation

Genomic DNA was extracted from cells fixed in acetic acid : methanol (1 : 3) by Puregene DNA Purification Kit (Gentra Systems, Minneapolis, MN, USA). DNA concentration was determined by a Nanodrop spectrophotometer. The quality of DNA was checked using agarose gel electrophoresis. DNA samples extracted from fixed cells of 2 healthy males and 2 healthy females by the same method were used as reference samples.

#### 2.3.3. Multiplex Ligation-Dependent Probe Amplification (MLPA)

The P377-A1 hematologic malignancies probemix and SALSA reagents were used for this study (MRC-Holland, Amsterdam, The Netherlands). Amplified probes and Genescan 500 ROX standard were separated by capillary electrophoresis using a 4-capillary ABI-PRISM 3130XL Genetic Analyzer (Applied Biosystems, Foster City, USA). Sizing of peaks and quantification of peak areas and heights were performed using GeneMarker v1.9 software (Applied Biosystems). A minimum of 4 healthy control samples were included in each run.

#### 2.3.4. High Resolution Array-Comparative Genomic Hybridization (aCGH)

aCGH was performed using Agilent SurePrint G3 Human Genome microarray 180 K (Agilent Technologies, Santa Clara, CA, USA), an oligonucleotide microarray containing approximately 180,000 probes 60-mer with a 17 kb average probe spacing. Genomic DNA of patient was cohybridized with a male control DNA (Agilent Technologies, Santa Clara, CA, USA). Labeling was performed using Agilent Genomic DNA enzymatic labeling kit (Agilent) according to the manufacturers' instructions. After hybridization, the aCGH slide was scanned on an Agilent scanner and processed with Feature Extraction software (v10.7) and results were analyzed using Cytogenomics (v2.9.1.3) using ADM2 as aberration algorithm.

## 3. Results of Retrospective Analysis

**Figure 1:**
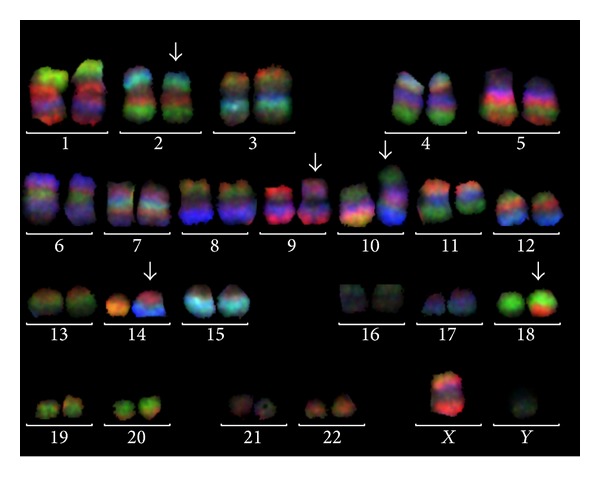
Application of mMCB showed no normal karyotype but derivative chromosomes 2, 9, 10, 14, and 18 (arrows). mMCB results are shown as overlay of three of the six used color channels. Evaluation was done as previously reported [10] using all 6 color channels and pseudocoloring. Breakpoints were determined as 2p23.2, 9p21.3, 10q24, 14q11, and 18q21.33.

**Figure 2:**
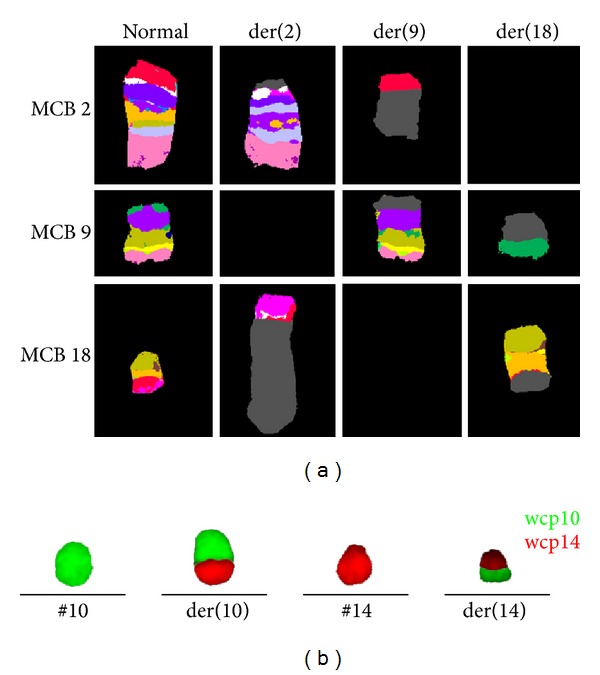
(a) Results of aMCB probe sets for chromosomes 2, 9, and 18 are shown in pseudocolor depiction, which confirmed the characterization of these three chromosomes involving rearrangement as t(2;9;18)(p23.2;p21.3;q21.33). (b) Whole chromosome paints (wcp) for chromosomes 10 and 14 confirmed that the t(10;14)(q24;q11) was independent of the t(2;9;18).

**Figure 3:**
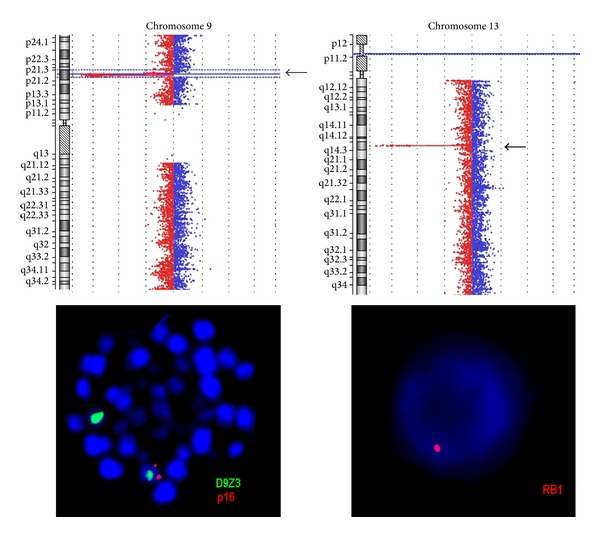
aCGH confirmed deletions in 9p21.3 and 13q14.2 (arrows) detected initially by MLPA (result not shown). FISH confirmed presence of these deletions in metaphase and/or interphase. Examples for heterozygote deletions of 9p21.3 and 13q14.2 are depicted; probes specific for the corresponding tumor suppressor genes were labeled in red; centromeric probe for chromosome 9 (D9Z3) was labeled in green.

As an initial test of retrospective analysis a genome wide FISH-banding applying mMCB was performed. Thereby, a previously unrecognized reciprocal and apparently balanced translocation between the three chromosomes 2, 9, and 18 was identified. Besides a known recurrent translocation of chromosomes 10 and 14 was recognized and the karyotype was suggested as 46,XY,t(2;9;18)(p23.2;p21.3;q21.33),t(10;14)(q24;q11) (Figure 1). aMCB and WCP probes as shown in Figure 2 confirmed these suggestions. Locus specific probes narrowed down the breakpoints as shown in Table 1(a). Unfortunately there was no sufficient cell pellet available to characterize the breakpoints in more detail than listed in Table 1(a). Even though closely located to the observed chromosomal breakpoints, direct involvement of the following oncogenes was excluded using locus specific FISH-probes for* ALK* in 2p23.2,* MLLT3* in 9p21.3, and* MALT1* and* BCL2* in 18q21.33. However, MLPA (result not shown) and aCGH (Figure 3) revealed that the t(2;9;18) is not really balanced: a deletion in 9p21.3 including* CDKN2A/INK4A/p16*,* CDKN2B/INK4B/p15*, and* MTAP/ARF/p14* could be found as chr9: 21,252,517–21,798,676x1 and 21,817,082–23,515,821x0 (hg19) (Figure 3; Table 1(b)). Moreover, a deletion in 13q14.2 was detected as chr13: 48,982,000–49,062,000x1 (hg19, Figure 3). FISH showed a mosaic condition of mixed heterozygous and homozygous deletion of 9p21.3 and 13q14.2 (Table 1(b)).

## 4. Discussion

Chromosomal translocations are considered to be the primary cause of leukemia for both acute and chronic phase. In this study, we retrospectively identified previously undetected balanced and unbalanced chromosomal and subchromosomal changes by application of molecular cytogenetics including FISH-banding, locus-specific FISH-probes, and aCGH plus MLPA. FISH-banding, especially mMCB, allows the identification of balanced and unbalanced inter- and intrachromosomal rearrangements of the whole human karyotype in one single experiment [10]. It might be indicated to apply mMCB or comparable FISH-banding approaches routinely in T-ALL cases exhibiting poor quality of the metaphase, that is, not well spreading ones with chromosomes appearing as fuzzy with indistinct margins [16, 17].

In this study one well-known and one yet unreported balanced translocation event were identified for a T-ALL as t(10;14)(q24;q11) and t(2;9;18)(p23.2;p21.3;q21.33), respectively. While a direct involvement of the cancer-related oncogenes* ALK* in 2p23.2,* MLLT3* in 9p21.3, and* BCL2* in 18q21.33 could be excluded, loss of two tumor suppresser gene loci in 9p21 and in 13q14 was found.

Data from the literature confirmed that the oncogenes tested and located nearby the chromosomal breakpoints of the three-way translocation were not yet found to be involved in T-ALL:* ALK* located in 2p23.2 was previously detected in a variety of B- and T-cell lymphomas and nonhematopoietic solid tumors [18–23], the* BCL2* gene is overexpressed in lymphomas [24, 25], and the* MLLT3* gene was one of the most highly upregulated transcripts and the most common fusion partner of* MLL* in* de novo* acute myeloid leukemia (AML) subtype M5 and therapy-related AML [26–28]; however, Meyer et al. [29] found that* MLLT3* also plays a role in pediatric rather than adult ALL.

In the present case, an additional chromosomal translocation t(10;14)(q24;q11), known as sole abnormality in 10% of T-ALL patients, was identified. Also it is present in 5% of pediatric and 30% of adult T-ALL [20, 30, 31]. The* TLX1* gene at 10q24 is a transcription factor becoming overexpressed as oncogene due to its juxtaposition to a strong promoter and enhancer elements of the TCR loci at 14q11 [5, 32–34]. A favorable outcome was reported in pediatric and adult T-ALL to be associated with the t(10;14) or TLX1 gene overexpression [5, 20, 35].

Even though balanced rearrangements are known to be typical for hematopoietic malignancies to date, only a limited number of studies have used whole genome directed FISH approaches to identify cryptic chromosomal abnormalities in ALL patients [36–38]. Still, in ALL it is uncommon to see three-way translocations. However, due to low metaphase resolution in ALL the real incidence of three-way translocations is currently unknown.

The present report highlights that after identification of apparently balanced chromosomal aberrations, it is still necessary to screen for further unbalanced submicroscopic abnormalities by molecular approaches such as MLPA and aCGH. However, also a confirmation of the results by molecular cytogenetics is necessary, as aCGH was partially misclassified a mix of homo- and heterozygote deletions as pure homozygote ones.

9p21.3 deletions, which lead to the loss of* CDKN2A/INK4A/p16*,* CDKN2B/INK4B/p15*, and* MTAP/ARF/p14* tumor suppressor genes expression, are the most predominant aberrations seen in precursor B-cell ALL (~20% of the cases) and T-ALL (>60% of the case) [39–42]. Besides also a deletion of* RB1* gene resulting in inactivation of another tumor suppressor gene expression was identified.* RB1* is rarely reported to be deleted in T-ALL. In contrast, deletion of* RB1* has been detected in 30% of B-ALL and nearly to 60% in B-CLL cases [43, 44]. Thus,* RB1* pathway was identified as potential targets for therapy of ALL [45, 46].

## 5. Conclusion

In conclusion, we report a case of T-ALL with complex chromosomal aberrations. Even if at time of diagnosis the deletion on 9p21.3 would have been detected and accordingly treated, it remains unclear what influence the other tumor suppressors and oncogenes (possibly) activated by the complex rearrangements would have had for the clinical outcome. Overall, the present case stresses the necessity to study hematological malignancies by different means to get a comprehensive picture of the genetic changes in connection with the acquired disease, as aCGH or MLPA alone would only have identified the imbalanced rearrangements, while molecular cytogenetics predominantly gave hints on the presence of balanced rearrangements.

## Figures and Tables

**(a):** 

Cytoband	Location [hg19]	Probe	Result for derivative chromosomes
2p24.3	chr2:	RP11-119F22	Signal on der(9); no split signal
	16,014,784–16,140,647		
2p23.3	chr2:	RP11-106G13	Signal on der(9); no split signal
	26,967,697–27,136,688		
2p23.2	chr2:	SPEC ALK	Signal on der(9); no split signal
	29,415,640–29,447,593		

9p22.1	chr9:	RP11-503K16	Signal on der(18); no split signal
	18,717,972–18,718,524		
9p22.1	chr9:	RP11-513M16	Signal on der(18); no split signal
	19,371,384–19,371,943		
9p21.3	chr9:	RP11-15P13	Signal on der(18); no split signal
	20,182,493–20,361,132		
9p21.3	chr9:	MLLT3	MLLT3-gene signal on der(18); no split signal
	20,344,968–20,621,872		
9p21.3	chr9:	SPEC p16	Deletion on der(9) and/or der(18)
	21,967,751–21,975,132		
9p21.3	chr9:	RP11-946B6	Deletion on der(9) and/or der(18) ish 9p21.3(RP11-946B6x0)[8]
	23,608,612–23,790,449		
9p21.2	chr9:	RP11-438B23	Signal on der(9); no split signal
	27,937,615–27,944,495		

18q21.32	chr18:	MALT1	MALT1-gene signal on der(18); no split signal
	56,338,618–56,417,370		
18q21	chr18:	BCL2	BCL2-gene signal on der(2); no split signal
	60,985,282–60,985,899		

**(b):** 

Cytoband	Location [hg19]	Probe	Result for derivative chromosomes
9p21.3	chr9: 21,967,751–21,975,132	SPEC p16	ish 9p21.3(p16x1)[4] nuc ish 9p21(p16x0)[64]/9p21(p16x1)[83]/ ** **9p21(p16x2)[53]

13q14.2	chr13: 48,920,000–49,140,000	LSI 13 = *RB1 *	nuc ish 13q14.2(*RB1*x0)[36]/ ** **13q14.2(*RB1*x1)[43]/ ** **13q14.2(*RB1*x2)[121]
